# The Role of Docosahexaenoic Acid in the Development of Preeclampsia and Perinatal Outcomes

**DOI:** 10.3390/diagnostics16020305

**Published:** 2026-01-17

**Authors:** Nalan Kuruca, Senol Senturk, Ilknur Merve Ayazoglu, Medeni Arpa, Mehmet Kagıtcı, Sibel Dogan Polat, Bülent Yılmaz

**Affiliations:** 1 Department of Obstetrics and Gynecology, Faculty of Medicine, Recep Tayyip Erdogan University, Rize 53100, Türkiye; dr.aates.9@gmail.com (N.K.); ilknurmervekazaz@gmail.com (I.M.A.); mehmetkagitci1@hotmail.com (M.K.); sibeldoganpolat@gmail.com (S.D.P.); drbulentyilmaz@gmail.com (B.Y.); 2 Department of Medical Biochemistry, Faculty of Medicine, Recep Tayyip Erdogan University, Rize 53100, Türkiye; medeni.arpa@erdogan.edu.tr

**Keywords:** preeclampsia, DHA, perinatal outcome, IUGR, NLR

## Abstract

**Background/Objectives**: Preeclampsia is a leading cause of maternal and perinatal morbidity worldwide, yet its underlying mechanisms remain unclear. Polyunsaturated fatty acids, particularly docosahexaenoic acid (DHA), are essential for placental development and vascular function, but evidence on their role in preeclampsia is inconsistent. This study aimed to compare serum DHA levels between women with preeclampsia and normotensive pregnant women and to examine their association with disease severity and maternal and perinatal outcomes. **Methods**: A total of 145 pregnant women aged 18–40 years were enrolled, including 47 with newly diagnosed preeclampsia (PE) and 98 normotensive controls. PE was defined according to the ACOG 2019 criteria. Serum DHA levels were measured using ELISA in fasting blood samples collected at the first visit. **Results**: Maternal serum DHA levels did not differ significantly between preeclampsia and control groups (*p* = 0.571); they were similar across control, mild PE, and severe PE groups. DHA showed a negative correlation with neutrophil-to-lymphocyte ratio (r = −0.305) and maternal hospitalization duration (r = −0.334). Independent predictors of PE included nulliparity (OR: 4.43), advanced age (OR: 1.14), elevated BMI (OR: 1.29), and low albumin (OR: 0.77). After adjusting for age and BMI, DHA was an independent negative predictor of IUGR (OR: 0.65). **Conclusions**: DHA levels: Placental and/or fetal DHA metabolism may be impaired in patients with preeclampsia. Although DHA was not associated with the development of PE, it was a negative predictor of IUGR. DHA reduces the length of maternal hospital stay through its anti-inflammatory effect.

## 1. Introduction

Preeclampsia (PE), which has no effective treatment and is characterized by systemic endothelial dysfunction, affects approximately 5–7% of all pregnancies and has the highest morbidity and mortality among hypertensive diseases of pregnancy [1,2]. Delays in diagnosis and treatment lead to an increased risk of maternal and fetal mortality, increased rates of cesarean delivery, maternal and neonatal intensive care unit admission, and preterm birth [3,4]. The American College of Obstetricians and Gynecologists (ACOG) defines PE as the presence of new-onset hypertension and proteinuria after 20 weeks of gestation in a previously normotensive patient [5]. In 2019, ACOG updated its 2013 guidelines [6]. According to this update, preeclampsia can be diagnosed in hypertensive pregnant women even in the absence of proteinuria if specific severe features are present, including elevated liver enzymes, thrombocytopenia, new-onset renal failure, pulmonary edema, or cerebral or visual symptoms [6]. Although delivery of the fetus and placenta is the only definitive treatment option for PE, it is associated with increased neonatal morbidity and mortality [2]. Although advancements in prenatal care and intensive care units are vital for the survival of both mothers and babies, PE remains a serious obstetric problem. Owing to these undesirable situations triggered by PE, research on the etiology and treatment of the disease continues unabated.

Inadequate placentation in the first trimester and increased antiangiogenic factors in the second and third trimesters cause uteroplacental ischemia, predisposing PE to become systemic [3]. Increased maternal genetic susceptibility [7] leads to increased oxidative stress, immunologic cell migration, inflammation, and endothelial dysfunction at the maternal–fetal interface, leading to the development of PE [8,9]. Polyunsaturated fatty acids (PUFAs), which are generally obtained through diet, contribute to the regulation of placental development and angiogenesis [10,11]. Docosahexaenoic acid (DHA), which is abundant in the placenta, is the most active form of PUFA and is converted to alpha-linolenic acid by fatty acid desaturase 2, supporting placental angiogensis [12]. Dietary intake of DHA, arachidonic acid, and eicosapentaenoic acid has been reported to prevent PE by promoting spiral artery remodeling [13]. Additionally, an experimental mouse study has shown that decreased DHA levels in trophoblasts impair placental angiogenesis and lead to PE-like symptoms [14].

The use of neutrophil-to-lymphocyte ratio (NLR), which is a biomarker of simple systemic inflammation, is increasing. Also, NLR is considered for its diagnostic, prognostic, and predictive potential in preeclampsia and pregnancy-related hypertensive disorders [15,16]. DHA levels in pregnancy, due to increasing lipid mobilization and placental transfer to the fetus, vary throughout trimesters [17]. Despite the fact that some studies demonstrate an inverse relationship between DHA levels and inflammatory markers, there has been no study which has investigated the association between DHA levels and NLR in preeclamptic pregnant women to the best of our knowledge. In this context, evaluation of the relationship between serum DHA levels and NLR in preeclamptic pregnancies may provide crucial insights into the inflammatory mechanisms underlying preeclampsia.

Despite the roles of PUFAs in placental development, it has not yet been clearly established whether serum DHA levels are affected in pregnant women with PE who do not receive PUFA supplements during pregnancy. This case–control study aimed to determine the differences in serum DHA levels in blood samples taken at the time of initial diagnosis in pregnant women with PE compared to those in healthy normotensive pregnant women. The relationships between DHA levels, PE severity, and maternal and perinatal outcomes were also analyzed.

## 2. Materials and Methods

This prospective cross-sectional study was conducted on pregnant women who visited the Department of Obstetrics and Gynecology of Recep Tayyip Erdoğan University Training and Research Hospital between October 2022 and June 2023. After the study was approved by the IRB of the same university (approval number: 2022/169; approval date: 29 September 2022), informed consent was obtained from all participants. Strict adherence to the ethical standards outlined in the Scientific Research and Publication Ethics Principles was maintained throughout the study. The sample size was determined using G*Power 3.1.9.7. At 80% statistical power and a significance level of α = 0.05, the smallest sample size was calculated as 138 (control: 94, study: 44), based on an effect size of d = 0.51 calculated using an independent-samples *t*-test. Given the possibility of missing data, 145 patients were included in this study. Accordingly, the study group consisted of 47 pregnant women with PE, and the control group consisted of 98 healthy, normotensive pregnant women. The inclusion criteria for the study group were patients aged 18–40 with a singleton pregnancy who are diagnosed with preeclampsia for the first time in their current pregnancy. The inclusion criteria for the control group were healthy pregnant women with no history of hypertension before or during pregnancy. In addition to demographic, clinical, and laboratory data, all participants were screened for epigastric pain, cerebral findings, and visual disturbances. Gestational age was determined based on the date of the last menstrual period, obstetric examination, and ultrasonography. BMI was calculated by dividing weight (in kilograms) by height (in meters) squared using a standard formula. For all participants, the mode of delivery, birth weight, whether the mother or baby was admitted to the intensive care unit, neonatal death, fetal anomaly, first and fifth minute APGAR scores, and length of hospital stay were recorded.

PE diagnosis was performed according to the 2019 diagnostic criteria of the American College of Obstetricians and Gynecologists (ACOG) [6]. Because the ACOG committee does not recommend routine screening for PE in pregnant women without a medical history or risk factors [6], further testing was performed only in women suspected of preeclampsia based on conventional follow-up tests and blood pressure (BP) measurements. Some patients were diagnosed with PE during outpatient follow-up, whereas others were hospitalized for several days. PE was defined as systolic blood pressure ≥ 140 mmHg or diastolic blood pressure ≥ 90 mmHg in measurements taken at least twice, 4 h apart, after the 20th week of gestation in a previously normotensive woman, accompanied by proteinuria. Proteinuria was defined as ≥300 mg of protein in a 24 h urine sample, a protein/creatinine ratio of ≥0.3, or 1 + proteinuria measured using a urine dipstick. Severe PE was diagnosed if the systolic BP was ≥160 mmHg and/or the diastolic BP was ≥110 mmHg. PE was diagnosed even in the absence of proteinuria if hypertension was accompanied by thrombocytopenia (platelets < 100,000/10^3^/μL), abnormal liver function tests (transaminases elevated to twice the normal value), new-onset renal failure (serum creatinine values > 1.1 mg/dL or a doubling of serum creatinine values in the absence of other renal diseases), pulmonary edema, or new-onset cerebral or visual disturbances.

A complete blood count, liver function tests, renal function tests, and complete urinalysis were performed at the time of admission. To prevent the potential effects of corticosteroid and MgSO_4_ treatments on laboratory parameters in pregnant women with PE, laboratory results obtained at initial admission or hospitalization were considered. Venous blood samples were collected between 8:00 and 10:00 AM following an overnight eight-hour fasting period. Samples were centrifuged at 4000 rpm for 10 min, separated into serum and plasma, and stored at −20 °C until analyzed. Biochemical analyses were performed spectrophotometrically using a Beckman Coulter AU680 Autoanalyzer (Beckman Coulter, Inc., Brea, CA, USA). Complete blood count analyses were performed using a Mindray BC6000 hematology analyzer (Mindray Medical International Limited, Shenzhen, China).

Women with a history of preeclampsia, stillbirth, chronic hypertension, chronic renal failure, pregestational diabetes, antiphospholipid syndrome, obesity, advanced maternal age, multiple gestation, and those who conceived using assisted reproductive technologies were excluded. Patients taking vitamin D and other vitamins, antioxidant nutritional supplements, insulin sensitizers, lipid-lowering drugs, aspirin, and low-molecular-weight heparin, which may directly or indirectly affect DHA levels, placental angiogenesis, and redox balance, were also excluded from the study. Pregnant women with molar pregnancies, those with a body mass index (BMI) > 30 kg/m^2^, and those diagnosed with rheumatologic, renal, or chronic cardiovascular diseases were also excluded.

While venous blood samples were collected for routine tests during outpatient clinic visits or hospitalizations, blood was also collected for DHA measurement, and the serum separated by centrifugation was stored at −20 °C until ELISA analysis. Serum DHA levels were quantified using ELISA (Cat. No: CEO623Ge, Cloud-Clone Corp, Wuhan, China). The kit manufacturer stated the analysis range as 12.35–1000 pg/mL and the analytical sensitivity as 5.16 pg/mL. The intra- and inter-assay coefficients of variation of the kit were <12%.

Statistical analyses were conducted using IBM SPSS Statistics version 27 (IBM Corp., Armonk, NY, USA). Graphical representations were generated using GraphPad Prism 10.4.2 software (GraphPad Software, San Diego, CA, USA). The normality of the data distribution was evaluated using the Kolmogorov–Smirnov test. Continuous variables adhering to the normality assumption are presented as mean ± standard deviation, whereas those not meeting this assumption are reported as medians (25th and 75th percentiles). Categorical data are expressed as frequency (*n*) and percentage (%). To compare parametric variables, an independent-samples *t*-test was employed. For non-parametric variables, the Mann–Whitney U or Kruskal–Wallis test was used, depending on the number of groups involved. Categorical variables were compared using the chi-square or Fisher’s exact test. Spearman’s correlation analysis was conducted to assess the relationship between DHA and other variables in patients with preeclampsia. A stepwise multivariable logistic regression analysis was conducted to identify significant independent predictors of preeclampsia. Furthermore, an independent association between the risk of intrauterine growth restriction (IUGR) and DHA levels in patients with preeclampsia was examined using binary logistic regression analysis, with adjustments made for age and body mass index (BMI). Statistical significance was set at *p* < 0.05.

## 3. Results

**Figure 1:**
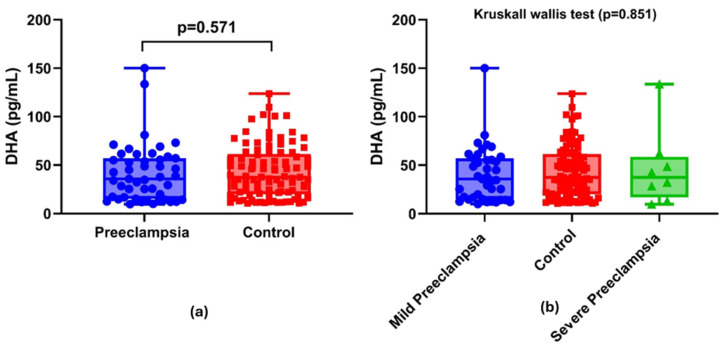
Comparison of maternal serum DHA concentrations: (**a**) DHA concentrations between preeclampsia and control groups; (**b**) DHA concentrations of mild preeclampsia, severe preeclampsia, and control groups.

The study comprised a control group of healthy pregnant women (*n* = 98) and a cohort of patients diagnosed with pre-eclampsia (*n* = 47), resulting in a total of 145 participants. Among patients with pre-eclampsia, 83% (*n* = 39) were classified as having mild pre-eclampsia, while 17% (*n* = 8) were identified as having severe preeclampsia. When these individuals diagnosed with preeclampsia were evaluated, cerebral visual impairment was detected in 19 of 47 patients (40.4%), right epigastric pain in 2 (4.3%), and pulmonary edema in 1 (2.1%). The 24 h urine protein excretion and spot urine protein-to-creatinine ratio (UPCR) in patients with preeclampsia were measured at 585 mg (296–2980 mg) and 0.43 mg/mg (0.21–1.46 mg/mg), respectively. The demographic and laboratory findings of healthy pregnant women and those with pre-eclampsia are shown in Table 1. There was no statistically significant difference between the preeclampsia and control groups in terms of mean age, gravida, and number of previous abortions (*p* > 0.05, all). However, the mean BMI was significantly higher in the preeclampsia group than that in the control group (*p* < 0.001). In this study, 55.3% of patients with preeclampsia (*n* = 26) were nulliparous, compared to 31.6% of the control group (*n* = 31). The incidence of nulliparity was significantly higher in the preeclampsia group than that in the control group (*p* = 0.011). In the preeclampsia group, there was a significant elevation in systolic blood pressure (SBP), diastolic blood pressure (DBP), neutrophils (NEUs), neutrophil-to-lymphocyte ratio (NLR), alanine aminotransferase (ALT), aspartate aminotransferase (AST), urea, creatinine, and C-reactive protein (CRP) levels compared to those in the control group (*p* < 0.05, all). Conversely, platelet (PLT) and albumin levels were significantly higher in the control group than in the preeclampsia group. No statistically significant difference was identified in the maternal serum DHA levels between the control group and patients with pre-eclampsia (*p* = 0.571) (Figure 1a and Table 1). The median DHA values for the control, mild preeclampsia, and severe preeclampsia groups were 37.45 (20.6–60.7), 35.8 (16–56.3), and 37.4 (20.7–55.3), respectively. No significant differences were observed among the three groups (*p* = 0.851) (Figure 1b).

The maternal and perinatal outcomes of the patients are presented in Table 2. In cases of preeclampsia, there was a significant increase in the duration of maternal hospitalization and preterm birth rates (*p* < 0.001 for each) compared with healthy pregnancies. The incidence of emergency cesarean sections was markedly higher in the preeclampsia group than in the control group, whereas the incidence of stimulated vaginal deliveries was significantly lower (*p* < 0.05 for each). Additionally, the birth weights of infants born to mothers with preeclampsia were significantly lower than those of infants in the control group (*p* < 0.001). There was a higher incidence of admission to the neonatal intensive care unit and fetal growth restriction in infants of mothers with preeclampsia (*p* < 0.001 and *p* = 0.005, respectively). No statistically significant differences were observed between the two groups in terms of Apgar scores at the first and fifth minutes. No neonatal deaths were reported in either group of the study.

Spearman correlation analysis identified significant negative correlations between maternal serum DHA concentration and both the neutrophil-to-lymphocyte ratio (NLR) (r = −0.305, *p* = 0.037) and the duration of maternal hospitalization (r = −0.334, *p* = 0.023) in patients with preeclampsia. No significant correlations were observed between maternal serum DHA concentrations and other variables (Table 3).

Multivariable logistic regression analysis revealed that nulliparity (OR: 4.433, 95% CI: 1.365–14.399, *p* = 0.013), advanced age (OR: 1.141, 95% CI: 1.018–1.279, *p* = 0.024), elevated BMI (OR: 1.292, 95% CI: 1.148–1.455, *p* < 0.001), and reduced albumin levels (OR: 0.773, 95% CI: 0.640–0.933, *p* = 0.007) were independently associated with preeclampsia. Other variables included in the analysis, such as DHA (*p* = 0.611), urea (*p* = 0.468), creatinine (*p* = 0.866), ALT (*p* = 0.929), AST (*p* = 0.239), NLR (*p* = 0.324), PLT (*p* = 0.259), and CRP (*p* = 0.780), were found to be non-significant (Table 4). Analysis of the significant variables revealed that nulliparity increased the odds of developing preeclampsia by a factor of 4.443. Additionally, an increase in age elevated the odds by a factor of 1.141, whereas a one-unit increase in body mass index (BMI) raised the odds by a factor of 1.292. Furthermore, a one-unit decrease in albumin level resulted in a 1.294-fold increase in the odds of developing preeclampsia.

When independent variables such as age and BMI were included in the model, DHA was found to have an independent negative predictive value for IUGR in patients with preeclampsia (OR: 0.653, 95% CI: 0.436–0.979; *p* = 0.039) (Table 5). After adjusting for age and BMI in patients with preeclampsia, a 1 pg/mL decrease in DHA levels increased the odds of IUGR by 1.531 times.

## 4. Discussion

Docosahexaenoic acid, which has both antioxidant and anti-inflammatory properties, is one of the most important PUFAs contributing to placental vasculogenesis [11,18]. While the omega-6/omega-3 PUFA ratio required for physiological cell development is 1, a ratio exceeding 10 in many dietary cultures is associated with an increased incidence of placental diseases, such as preeclampsia [19]. Although preeclampsia is a multifactorial disease, it is associated with increased inflammation, oxidative stress, and impaired PUFA transport [11,19,20]. In PE, a defect in DHA metabolism or transport may impair placental angiogenesis, leading to placental malperfusion and disease initiation or progression [2,10]. However, to date, a clear causal relationship between maternal serum DHA levels and PE has not been established [14].

In the present study, serum DHA levels in blood samples taken at the time of PE diagnosis were compared with those of normotensive pregnant women, and no differences were found. When we divided the preeclamptic patients into mild and severe groups, we also found no difference in serum DHA levels. The results of previous studies on serum DHA levels in patients with PE are heterogeneous. While most studies reported a decrease in serum DHA levels [21,22], some studies emphasized the importance of a decrease in placental DHA levels rather than serum DHA [14]. We did not measure placental DHA levels, which limits our ability to make definitive statements on this matter. However, placental DHA deficiency may trigger the development of PE, even if serum DHA levels are sufficient. The demonstration that fatty acid desaturase 2 expression, a rate-limiting enzyme in fatty acid desaturation, is reduced in the placentas of patients with PE, independent of maternal DHA levels, supports our hypothesis [23]. Consistent with this, Liu et al. [14] reported that placental DHA deficiency may contribute to the development of PE by disrupting the signaling pathways required for placental vascularization. In conclusion, although DHA plays a role in fetal and perinatal development, we cannot claim that it is a predictor of PE based on serum levels alone. In accordance with the last statement, DHA concentrations in the placental intervillous space are three to four times higher than those in the maternal circulation, demonstrating that serum DHA levels do not reflect placental levels [24].

Despite similar serum DHA levels in pregnant women with PE and those who are normotensive, the higher rates of neonatal intensive care unit admission, fetal growth restriction, and preterm birth in the PE group suggest that PE causes a defect in DHA transport or function. The negative association between maternal serum DHA concentration and length of hospital stay suggests that adequate maternal serum DHA levels may prevent maternal complications of PE. If DHA had reached adequate levels in the placenta and/or fetus, there might not have been an increase in NICU admission, fetal growth restriction, or preterm birth rates compared to those in healthy pregnant women. Therefore, normal maternal serum DHA levels in patients with PE should not be interpreted as indicating functional placental or fetal DHA pathways. The negative association between maternal serum DHA concentration and the neutrophil-to-lymphocyte ratio may indicate that DHA, through its anti-inflammatory effect, improves the maternal compartment of the chronic inflammatory state in PE. The high rates of NICU admission, fetal growth restriction, and preterm birth in PE despite normal maternal serum DHA levels may reflect a deficiency or dysfunction of DHA at the fetal or placental levels.

Multivariate regression analysis revealed that nulliparity, advanced maternal age, high body mass index, and low serum albumin levels were independently associated with preeclampsia. However, a significant association was found only between maternal serum DHA levels and IUGR incidence. After adjusting for independent variables such as age and BMI, DHA had an independent negative predictive value for IUGR in patients with preeclampsia. Every 1 pg/mL decrease in maternal serum DHA levels led to a 1.5-fold increase in the odds of IUGR. Although the body has evolved to meet the DHA requirement critical for fetal development, the fact that DHA is a negative predictor of IUGR requires clarification. Under physiological conditions, gestational hyperlipidemia is an important mechanism that facilitates placental fatty acid transport and ensures adequate DHA availability to the fetus [11]. The placenta ensures the prioritized and timely delivery of adequate DHA from the mother to the fetus [18]. Preeclampsia and other placental diseases can compromise DHA availability in the fetus [25]. Measuring fetal cord and placental DHA levels may help address this question. Although PE development is influenced by maternal age, serum albumin levels, BMI, and maternal age, the lack of a correlation with DHA is not a paradox. The primary function of DHA is not to regulate placental formation but to regulate fetal neurodevelopment [18,25]. Although the development of the placenta, a critical organ for the fetus, is influenced by the parental genome, fatty acids in the maternal circulation, cytokines, and angiogenic factors, a decrease or increase in a single molecule is not a determinant of placental formation [20]. Therefore, the fact that DHA is a negative predictor of IUGR but not associated with PE is consistent with the physiological properties of the molecule.

While DHA supplementation has been reported to improve fetal birth weight [26], we found that fetal birth weight was lower in pregnant women with PE than in normotensive pregnant women. Low fetal birth weight despite normal serum DHA levels may indicate that DHA alone does not improve placental malperfusion associated with PE. Dietary DHA intake is recommended for the development of the fetal brain and visual function [26]. However, despite normal serum DHA levels, the lower fetal birth weight observed in pregnancies complicated by preeclampsia suggests that low birth weight in PE may be more closely related to a possible placental transport dysfunction rather than to serum DHA levels.

The lack of a significant association between serum DHA levels and perinatal outcomes is consistent with previous studies, suggesting a complex, multifactorial relationship between maternal PUFA levels and fetal outcomes [13]. Despite low serum DHA concentrations in pregnant women with PE, the high DHA levels in breast milk provide further evidence that DHA in the maternal circulation does not reflect placental, milk, or fetal DHA levels [27]. The higher DHA levels in milk than in serum suggest that the body may direct DHA to different organs according to its needs and changes in physiological conditions. The lack of association between serum DHA levels and perinatal outcomes may be due to the timely and adequate delivery of DHA to the target site.

The similarity in serum DHA levels between patients with severe and mild PE suggests no direct relationship between DHA and PE development. The limited number of studies investigating PE severity and DHA levels have produced heterogeneous results. One study reported an increase in total PUFA concentrations and a decrease in DHA percentage in women with severe preeclampsia [28]. Another study reported similar omega-3 and omega-6 fatty acid profiles in patients with mild and severe PE [29]. One study reported an inverse association between DHA intake and the risk of preeclampsia [13], while another reported a 33% lower risk of severe preeclampsia with DHA supplementation during pregnancy [30]. However, no study has clearly documented the association between DHA and the development of severe PE.

It is important to emphasize the limitations of this study. Plasental or fetal DHA levels could not be measured, which can be stated as a major limitation of the study. Assessing DHA levels in maternal serum, cord blood, and placental tissue could provide more information about the role of DHA in PE. Although the sample size was sufficient for statistical analysis, the current number of participants limits the generalizability of our findings. The cross-sectional design of the study and the analysis of only a single blood sample at the first visit make it difficult to establish a causal relationship between DHA and PE. Finally, the single-center design may limit the applicability of the results to broader populations and different healthcare settings.

## 5. Conclusions

The similarity of serum DHA levels between women with PE and normotensive pregnant women and the lack of a relationship between DHA and PE severity suggest that the role of serum DHA levels in the etiology of PE and placental malperfusion is limited. PE may disrupt placental and/or fetal DHA metabolism despite normal serum DHA levels. Although DHA was not associated with the development of PE, it was a negative predictor of IUGR. DHA reduces inflammation and shortens the duration of maternal hospitalization. Longitudinal studies analyzing DHA in blood samples collected after PE diagnosis and at the time of delivery may allow us to draw more definitive conclusions regarding the role of PUFAs in the etiology of PE.

## Figures and Tables

**Table 1:** Demographic and laboratory characteristics of the control and preeclampsia groups.

	Control Group (*n* = 98)	Preeclampsia Group (*n* = 47)	*p*-Value
Age (year)	29.37 ± 5.06	31.15 ± 5.15	0.051 ^a^
BMI (kg/m^2^)	28.75 ± 5.11	34.01 ± 5.44	<0.001 ^a^
Previous abortions	0 (0–1)	0 (0–1)	0.596 ^b^
Gravida	2 (1–4)	2 (1–3)	0.059 ^b^
Nulliparity (*n*, %)	31 (31.6)	26 (55.3)	0.011 ^c^
SBP (mmHg)	110 (100–120)	150 (140–150)	<0.001 ^b^
DBP (mmHg)	70 (60–75)	90 (90–98)	<0.001 ^b^
NEU count (10^9^/L)	7.32 ± 1.76	8.50 ± 2.64	0.007 ^a^
LYM count (10^9^/L)	1.84 (1.55–2.11)	1.75 (1.40–2.18)	0.447 ^b^
NLR	3.89 (3.27–4.75)	4.65 (3.34–5.77)	0.023 ^b^
PLT count (10^9^/L)	254.72 ± 70.57	229.25 ± 60.6	0.035 ^a^
ALT (U/L)	10 (8–13)	12 (9.5–14)	0.020 ^b^
AST (U/L)	15 (12–18)	17 (14–23.5)	0.008 ^b^
Urea (mg/dL)	14 (11–16)	19 (14–24)	<0.001 ^b^
Creatinine (mg/dL)	0.48 (0.43–0.52)	0.51 (0.45–0.59)	0.015 ^b^
Albumin (g/L)	35.48 ± 1.81	32.5 ± 4.1	<0.001 ^a^
CRP (mg/L)	5.9 (3.3–9.7)	8.6 (6.4–13.4)	0.003 ^b^
DHA (pg/mL)	37.45 (20.6–60.7)	35.8 (16–56.3)	0.571 ^b^

^a^ Student’s *t*-test; ^b^ Mann–Whitney U test; ^c^ Chi-square test. ALT: alanine aminotransferase; AST: aspartate aminotransferase; BMI: body mass index; CRP: C-reactive protein; DHA: docosahexaenoic acid; LYM: lymphocyte; NEU: neutrophil; NLR: neutrophil-to-lymphocyte ratio; PLT: platelet; SBP: systolic blood pressure; DBP: diastolic blood pressure.

**Table 2:** Maternal and perinatal outcomes among control and preeclamptic women.

		Control Group (*n* = 98)	Preeclampsia Group (*n* = 47)	*p*-Value
Mode of delivery	Stimulated vaginal	33 (33.7%)	5 (10.6%) *	
	Induced vaginal	2 (2%)	2 (4.3%)	0.007 ^d^
	Emergency cesarean	36 (36.7%)	28 (59.6%) *	
	Elective cesarean	27 (27.6%)	12 (25.5%)	
Gestational age (*n*, %)	Preterm	4 (4.1%)	16 (34.0%)	<0.001 ^c^
	Term	94 (95.9%)	31 (66.0%)	
Birth weight (g)		3316.1 ± 373.15	2651.02 ± 686.7	<0.001 ^a^
Fetal growth restriction (*n*, %)		3 (3.1%)	8 (17%)	0.005 ^d^
NICU admission (*n*, %)		0 (0.0%)	11 (%23.4)	<0.001 ^d^
Apgar score at 1 min		8 (8–9)	8 (8–9)	0.226 ^b^
Apgar score at 5 min		9 (9–10)	9 (9–10)	0.154 ^b^
Postpartum hospital stay (day)		1 (1–2)	3 (3–5)	<0.001 ^b^

^a^ Student’s *t*-test; ^b^ Mann–Whitney U test; ^c^ Chi-square test; ^d^ Fisher’s exact test. * *p* < 0.05 vs. control group. NICU: neonatal intensive care unit.

**Table 3:** Correlation analysis results between maternal serum DHA levels and other parameters in PE.

	r	*p*-Values
Age (years)	−0.150	0.316
BMI (kg/m^2^)	−0.030	0.843
SBP (mmHg)	0.137	0.359
DBP (mmHg)	−0.077	0.609
NLR	−0.305	0.037
PLT (109/L)	−0.033	0.824
ALT (U/L)	0.092	0.539
AST (U/L)	0.098	0.514
Urea (mg/dL)	−0.057	0.563
Creatinine (mg/dL)	−0.214	0.149
Albumin (g/L)	0.065	0.664
Proteinuri (mg/24 s)	0.243	0.348
UPCR (mg/mg)	0.335	0.057
CRP (mg/L)	−0.256	−0.082
Apgar score at 1 min	0.245	0.113
Apgar score at 5 min	0.240	0.121
Postpartum hospital stay in days	−0.334	0.023
Birth weight (g)	−0.070	0.652
Gravida	−0.012	0.938

r: Spearman correlation coefficient. ALT: alanine aminotransferase; AST: aspartate aminotransferase; BMI: body mass index; CRP: C-reactive protein; NLR: neutrophil-to-lymphocyte ratio; PLT: platelet; SBP: systolic blood pressure; DBP: diastolic blood pressure; UPCR: spot urine protein-to-creatinine ratio.

**Table 4:** Results of multivariate logistic regression analysis.

	*p*-Values	OR	95% CI	
			Lower	Upper
Age	0.024	1.141	1.018	1.279
Body mass index	<0.001	1.292	1.148	1.455
Nulliparity	0.013	4.433	1.365	14.399
Urea	0.468	1.018	0.970	1.068
Creatinine	0.866	1.724	0.003	9.535
ALT	0.929	0.994	0.869	1.137
AST	0.239	1.083	0.948	1.237
Albumin	0.007	0.773	0.640	0.933
DHA	0.611	0.995	0.977	1.014
NLR	0.324	1.222	0.821	1.819
PLT	0.259	0.995	0.987	1.004
CRP	0.780	1.013	0.926	1.108

OR: odds ratio; CI: confidence interval; CRP: C-reactive protein; ALT: alanine aminotransferase; AST: aspartate aminotransferase; DHA: docosahexaenoic acid; NLR: neutrophil-to-lymphocyte ratio; PLT: platelet.

**Table 5:** Serum DHA levels independently associated with intrauterine growth restriction in preeclampsia patients, results of binary logistic regression analysis (*n* = 47).

	Unadjusted		Adjusted ^(1)^	
	OR (95% CI)	*p*	OR (95% CI)	*p*
DHA	0.797 (0.642–0.988)	0.039	0.653 (0.436–0.979)	0.039

OR: Odds ratio. CI: Confidence interval. ^(1)^ Adjusted with age, body mass index.

## Data Availability

The original contributions presented in this study are included in the article. Further inquiries can be directed to the corresponding author.

## Abbreviations

The following abbreviations are used in this manuscript:

DHA	Docosahexeenoic Acid
ACOG	American College of Obstetricians and Gynecologist
PE	Preeclampsia
PUFA	Polyunsaturated Fatty Acids
UPCR	Urine protein-to-creatinine ratio
NICU	Neonatal İntensive Care Unit
IRB	Institutional Review Board
